# Anesthetic Challenges Posed by Heavy Kratom Users

**DOI:** 10.7759/cureus.22864

**Published:** 2022-03-05

**Authors:** Elisa Lund, Aaron B Low, Jennifer D Allan, Jose A Puentes, David N Flynn

**Affiliations:** 1 Anesthesiology, University of North Carolina, Chapel Hill, USA; 2 Pediatric Anesthesiology, University of North Carolina, Chapel Hill, USA

**Keywords:** alpha-2 agonism, withdrawal, opioids, substance use, pain management, anesthesia, kratom

## Abstract

Kratom is a herbal and natural dietary supplement from Southeast Asia that is gaining popularity in the United States. Its leaves contain multiple psychoactive chemicals that stimulate opioid, alpha-2, and serotonergic receptors. Kratom is used as a stimulant and in the treatment of anxiety, pain, and opioid withdrawal. In most states, kratom can be purchased legally and is sold at smoke shops, gas stations, and online. To date, only limited data is available on the impact of habitual kratom use on patients undergoing anesthesia. The following case report highlights multiple anesthetic challenges posed by a heavy kratom user.

## Introduction

Kratom (*Mitragyna speciosa*) is a medicinal plant indigenous to Southeast Asia where it is used as a stimulant, analgesic, and in the treatment for opioid withdrawal and addiction [1-3]. Traditionally, kratom leaves were consumed either wholly or as tea, although they can be chewed, crushed, smoked, powdered, placed in capsules, or concentrated into extracts [2].

Kratom is gaining popularity in the United States, where the estimated annual prevalence of use is 0.8% [4]. It can be purchased from online retailers and is also sold in smoke shops, gas stations, and supplement stores [2]. Although it has no FDA-approved uses, it is promoted as a safe herbal supplement to treat pain, anxiety, fatigue, and opioid addiction [2]. However, its use can lead to dependency, addiction, and abuse as well as serious side effects, including respiratory depression, seizures, arrhythmias, and death [5,6]. At least 90 overdose deaths were attributed to kratom between 2016 and 2017 [6]. Nevertheless, kratom remains legal throughout much of the United States, excluding six states: Alabama, Arkansas, Indiana, Rhode Island, Vermont, and Wisconsin [7].

The pharmacology of kratom is complex. For oral ingestion, low doses (1-5 g) produce stimulant effects, while higher doses (5-15 g) induce opioid-like effects with sedation [6]. Although at least 40 alkaloids have been isolated from kratom leaves, the primary psychoactive substances in kratom are mitragynine and 7-hydroxymitragynine [5]. Analysis of kratom alkaloids suggests ingestion results in the stimulation of opioid, alpha-2, and serotonin receptors [8]. With increasing popularity in the United States, anesthesiologists must understand how kratom use affects perioperative management. To our knowledge, however, only two case reports have been published reporting perioperative management of kratom users, with both reporting unremarkable perioperative courses [9,10]. In this report, we describe the anesthetic challenges encountered while caring for a heavy kratom user.

## Case presentation

An 18-year-old, 52-kg male with a history of bilateral idiopathic condylar resorption causing retrognathia and sleep apnea presented for LeFort I osteotomy with bilateral fifth rib resection, bilateral mandibular bone grafts, and genioplasty with genioglossus advancement. He had no other medical issues and had not taken any medications. His vital signs were within normal limits. A physical examination revealed micro- and retrognathia and a Mallampati class II airway with a good mouth opening. His cardiac and pulmonary examinations were normal. He had a history of substance use, including kratom several times daily (up to 35 g/day), occasional marijuana, and rare lysergic acid diethylamide (LSD) use. He had used kratom the evening before surgery, marijuana one week before surgery, but had not used LSD for several months.

Preoperatively, the patient received 2 mg IV midazolam. Induction of anesthesia was notable for resistance to anesthetics. Despite receiving 300 mg of propofol (6 mg/kg), 150 mcg of fentanyl citrate (3 mcg/kg), and 60 mg of lidocaine, general anesthesia had not been achieved. He was then mask ventilated for several minutes with 4-6% inhaled sevoflurane. After loss of consciousness, intubation was facilitated with 60 mg of succinylcholine. Anesthesia can be maintained with infusions of propofol (150-200 mcg/kg/min) and remifentanil (0.15-0.2 mcg/kg/min), titrated to Bispectral Index^TM^ (BIS^TM^; Covidien Medical, Boulder, CO) reading of 50.

Upon surgical incision, the patient’s mean arterial blood pressure increased from 60 to 110 mmHg. Boluses of dexmedetomidine and fentanyl were administered with minimal improvement. Subsequently, infusions of nicardipine (5-12.5 mg/h) and esmolol (50-200 mcg/kg/min) were required to achieve a mean arterial pressure of less than 80 mmHg. Both infusions were continued until the completion of surgery.

During the 9-h surgery, the patient received a total of 5,400 mg of propofol, 5.5 mg of remifentanil, 3,200 mg of esmolol, 19 mg of nicardipine, 52 mcg of dexmedetomidine in divided boluses, 400 mcg of fentanyl, 2 mg of morphine, 800 mg IV acetaminophen, and bupivacaine infiltration of the surgical sites by the surgeons. Before emergence, a thoracic epidural was placed in the operating room loaded with 5 ml of 1.5% lidocaine with 1:200,000 epinephrine and 0.125% bupivacaine infusion initiated at a rate of 7 ml/h.

The emergence of anesthesia was complicated by extreme pain, agitation, and aggression. The agitation worsened in the post anesthesia care unit (PACU), prompting concerns for emergence delirium. The attending anesthesiologist remained at the patient’s bedside in the PACU and administered additional boluses of propofol (20 mg), fentanyl (25 mcg), dexmedetomidine in 4 mcg aliquots (20 mcg), ketorolac (25 mg), and lorazepam (1 mg IV). When the delirium was resolved, his thoracic epidural was verified to cover the thoracic incision site. However, he reported a pain score of 10/10 using the NRS-11 pain scoring system at his mandibular incision and received 5 mg of oral oxycodone solution. Despite his reported pain score only improving to 8/10, he was discharged to the pediatric inpatient ward. His inpatient pain medications included 10 ml of oral hydrocodone-acetaminophen (7.5 mg/325 mg per 15 ml) every 4-6 h as needed for pain.

At 2 am on the morning after surgery, the patient’s thoracic pain had resolved but he complained of 10/10 jaw pain, prompting consultation with the chronic pain team. His refractory pain was attributed to opioid resistance secondary to habitual kratom use. Hydrocodone-acetaminophen was held, and an IV hydromorphone patient-controlled analgesic pump (0.3 mg every 10 min) was initiated. Additionally, he was administered gabapentin 600 mg every night and ibuprofen 600 mg every 6 h. Four hours later, after receiving 1.2 mg of hydromorphone, the patient-controlled analgesia was discontinued, and he was transitioned back to hydrocodone-acetaminophen. He subsequently received five doses of hydrocodone-acetaminophen (10 ml/dose) per day on post-operative days (POD) 1 and 2. Patient-reported pain scores ranged from 6-9/10 on POD1 and 5-9/10 on POD2. Despite continued discomfort, the patient and family felt his pain regimen was acceptable and his medical team deemed him suitable for discharge. He was discharged home on the evening of POD2. His discharge analgesic regimen included ibuprofen 600 mg every 6 h as needed, gabapentin 600 mg at night, and 10 ml of hydrocodone-acetaminophen (7.5 mg/325 mg per 15 ml) every 4 h as needed for pain.

## Discussion

**Figure 1:**
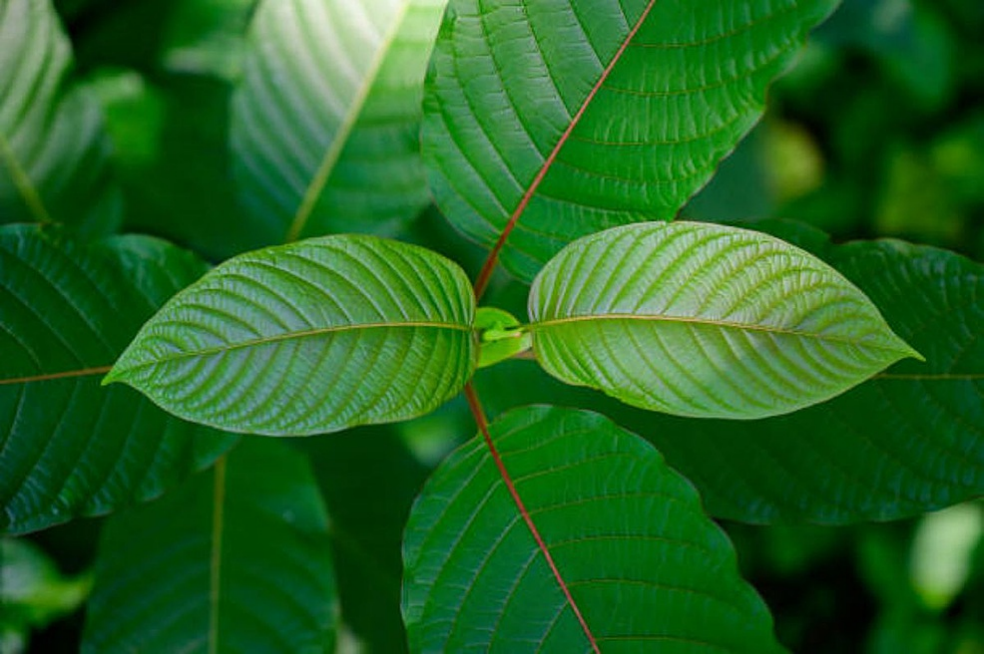
Kratom is derived from M. speciosa, a plant from Southeast Asia Publicly sourced photograph. Accessed at gettyimages.com November 20, 2021.

**Figure 2:**
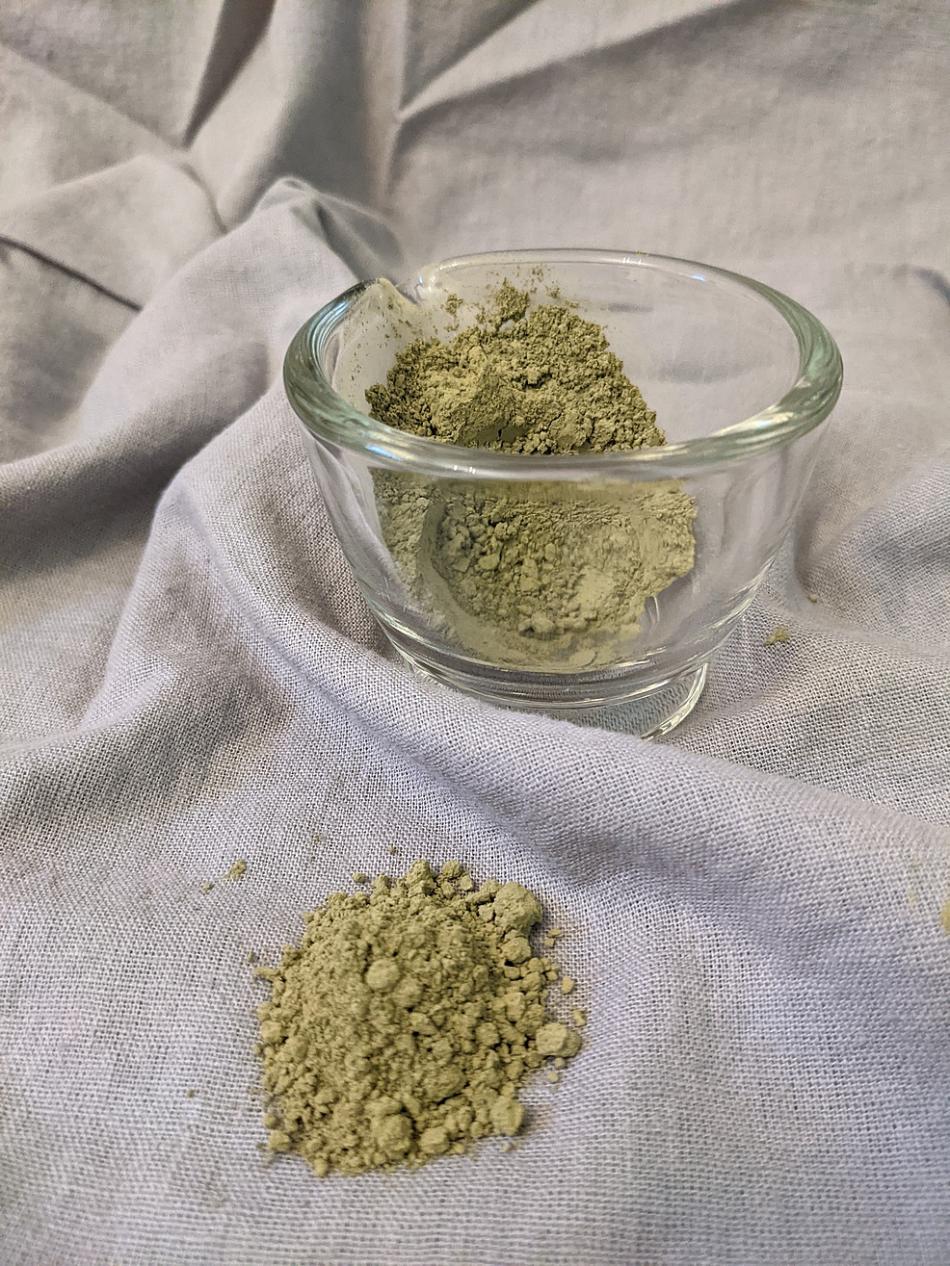
Kratom as a crushed powder for consumption. It can be chewed, crushed, smoked, powdered, placed in capsules, or concentrated into extracts Photograph credit, Elisa Lund.

Kratom is an increasingly popular, unregulated herbal product that can be legally purchased in most states (Figures 1-2). Kratom leaves contain dozens of chemically active compounds that interact with opioid, alpha-2, and serotonin receptors, resulting in stimulation at low doses and opioid-like effects at high doses. The patient in this case report was a heavy kratom user, consuming approximately 35 g/day. Several aspects of his perioperative course were notable.

First, the patient exhibited resistance to IV anesthetics. Despite receiving 2 mg IV midazolam, followed by approximately 6 mg/kg of propofol and 3 mcg/kg of fentanyl, an inhaled anesthetic was required to achieve a state of general anesthesia. Habitual, high-dose kratom abuse likely contributed to the observed anesthetic resistance, due to the upregulation of receptors in the central nervous system.

Second, the patient exhibited tolerance to opioids and poorly controlled pain. Intraoperatively, the patient developed refractory hypertension immediately following surgical incision, despite receiving remifentanil at 0.2 mcg/kg/min and additional boluses of fentanyl and dexmedetomidine. Infusions of nicardipine and esmolol were required for blood pressure control, both of which are rarely used in our institution. Stable BIS^TM^ readings of approximately 50 indicate the adequate depth of anesthesia and therefore the hypertension was likely a manifestation of uncontrolled intraoperative pain due to kratom-induced opioid resistance.

Postoperatively, the patient continued to suffer from poorly controlled pain, despite the placement of an epidural and consultation of the chronic pain team. However, the patient did not complain of thoracic pain while the epidural infusate covered the thoracic incision, suggesting that neuraxial and regional anesthesia should be implemented when appropriate for patients who use kratom. Unfortunately, he continued to experience significant jaw pain that was poorly controlled despite multimodal therapy with scheduled ibuprofen (600 mg every 6 h), scheduled gabapentin (600 mg every night), acetaminophen, and opioids (as 10 ml of hydrocodone-acetaminophen every 4 h as needed for pain).

Third, the patient exhibited delirium during emergence from anesthesia. His delirium may have been related to stress from uncontrolled pain. However, it is also possible that the delirium was a manifestation of kratom withdrawal. Kratom withdrawal can begin the day following cessation and include both physical (e.g., pain, nausea, tremors) and psychological (e.g., anger, hostility, aggression) symptoms [7,8]. Kratom withdrawal has been treated with clonidine [9], opioids [10], and benzodiazepines [11]. In the PACU, the patient’s agitation improved after he received dexmedetomidine, lorazepam, fentanyl, and oxycodone. Thus, it is possible that the anesthesiologist inadvertently but appropriately treated kratom withdrawal with this combination of medications.

Currently, there are no guidelines for the perioperative management of patients who use kratom. However, given the potential impact of kratom use on anesthetic management, we recommend asking patients about supplement use during the preoperative interview. Kratom users should be advised that their anesthetic course could be unpredictable and that pain management may be challenging. Table 1 describes the anesthetic challenges encountered with this patient and the potential interventions anesthesiologists can take when caring for heavy kratom users. Postponement of surgery should also be considered for patients who consume high doses of kratom regularly. It is sensible to advise patients to wean from kratom before surgery, although guidance from an addiction specialist may be required given the possibility of severe withdrawal symptoms by heavy users. Multimodal analgesia should be employed and regional and neuraxial anesthesia should also be used when possible.

**Table 1:** Anesthetic challenges in a patient with heavy kratom use BIS^TM^, Bispectral Index^TM^;^ ^EEG, electroencephalogram.

	Challenge encountered	Proposed mechanism	Suggested intervention
Anesthetic induction	Resistance to intravenous anesthetics	Receptor modulation due to chronic activation by kratom alkaloids	Anticipation of potential for unusually high doses. Consider the use of EEG/BIS^TM ^to monitor anesthetic depth
Anesthetic maintenance	Refractory hypertension	Inadequate pain control due to kratom-induced opioid resistance	Intraoperative EEG monitoring/BIS^TM^ to monitor anesthetic depth. Multimodal approach to pain control. Use regional/neuraxial anesthesia if possible. Consider arterial line for hemodynamic monitoring
Anesthetic emergence	Severe emergence delirium	Poorly controlled pain. Possible kratom withdrawal	Multimodal approach to pain control. Optimal treatment for withdrawal unclear (consider alpha-2 agonists, opioids, benzodiazepines)
Postoperative pain control	Poorly controlled pain	Kratom-induced opioid resistance	Multimodal approach to pain control, including use of regional/neuraxial anesthetics. Anticipate higher-than-normal opioid requirements. Consider chronic pain consult
When possible, advise tapering or cessation of kratom use before surgery. For heavy, habitual, or long-term users, consider consultation with an addiction specialist for assistance with tapering. Abrupt cessation can lead to serious withdrawal symptoms			

## Conclusions

With the increasing use of kratom in the United States, anesthesiologists must understand the potential implications of habitual kratom use on patients receiving anesthesia. The patient in this case report was a heavy kratom user. His perioperative course was complicated by resistance to IV anesthetics and opioids, poorly controlled pain, and emergence delirium. It is possible that some of his symptoms were secondary to kratom withdrawal along with opioid resistance from kratom's opioid receptor agonism. Patients with heavy kratom use may require higher narcotic doses, multimodal analgesia, and neuraxial or regional anesthesia. Given the challenges posed by habitual kratom users, patients who abuse kratom should be advised to wean or stop kratom use before surgery.
